# Relationship between patient-reported outcome measures (PROM) and three measures of foot–ankle alignment in patients with metatarsal head pain: a cross-sectional study

**DOI:** 10.1186/s13047-016-0182-1

**Published:** 2016-12-16

**Authors:** Manuel González-Sánchez, Esther Velasco-Ramos, Maria Ruiz Muñoz, Antonio I Cuesta-Vargas

**Affiliations:** 1 Departamento de Fisioterapia, Universidad de Málaga, Instituto de Investigación Biomédica de Málaga (IBIMA) (Grupo de Clinimetría FE-14), Malaga, Spain; 2 Universidad de Málaga, Departamento de Enfermería y Podología, Instituto de Investigación Biomédica de Málaga (IBIMA) (Grupo de Clinimetría FE-14), Málaga, Spain; 3 Facultad de Ciencias de la Salud, Universidad de Málaga, Malaga, Spain; 4 School of Clinical Sciences at Queensland University, Brisbane, Australia

**Keywords:** Metatarsalgia, Outcome assessment, Quality of life, Measurement, Questionnaires, Regression analysis

## Abstract

**Background:**

The aim of the present study is to establish the relationship between foot–ankle patient-reported outcome measures (PROM) and three measures of foot–ankle alignment (MoFAA) in patients with metatarsal head pain.

**Methods:**

A cross-sectional study where 206 patients completed three PROMs and a clinician recorded three MoFAA bilaterally (three times each). A reliability analysis of the MoFAA, a correlation analysis (between MoFAA and PROM) and regression analysis (dependent variable: PROM; independent variables: MoFAA) were performed.

**Results:**

Pearson’s coefficient changed in each PROM used, ranging from 0.243 (AAOS-FAM_ShoeComfortScale_–FVA_Right_) to 0.807 (FFI_Index_–first MTPJE_right_). Regression indices (R^2^-corrected) ranged between 0.117 (AAOS-FAM_ShoeComfortScale_) and 0.701 (FFI_Index_).

**Conclusions:**

The MoFAA correlated between moderately to strongly with the foot–ankle PROM selected. The level of correlation between MoFAA and PROM was higher when patients with metatarsal head pain were asked about foot health status, pain and function; however, the correlation was poor when the patient was asked about shoe aspects. In addition, the MoFAA variable that achieved the highest correlation value was the first metatarsophalangeal joint extension. The results obtained in this study could be used in future studies to develop tools for assessing and monitoring patients with metatarsal head pain.

## Background

About 20% of people over 65 have non-traumatic foot problems, and 60% of these problems are localized in the forefoot [1]. Among all the pathologies suffered by the forefoot, metatarsal head pain is the leading cause of foot consultation [2].

There are two options for patient evaluation and monitoring: objective clinical outcome measures (OCOM) [3] and patient-reported outcome measures (PROM) [4]. The OCOM are objective tests that provide a reliability and validity degree, and promote trust in results [5]. In contrast, PROM are used worldwide in daily clinical practice and research as a way to quantify a patient’s perception of disability, health and quality of life [6]. OCOM and PROMs help normalize results, reduce errors and improve understanding of results for both patients and clinicians [3].

From a biomechanical approach, the first ray is a key element in controlling the structural integrity of the foot [7], facilitating forward progression during walking [8] and generating the windlass mechanism [8]. Biomechanical disorders of the first ray are considered a critical factor of several pathologies of the feet [7]. In addition, in adults, hindfoot valgus is associated with both Hallux valgus [9, 10] and Hallux limitus/rigidus [9]. In clinical practice, three measures of foot–ankle alignment (MoFAA) are frequently used in the assessment and monitoring of patients with foot–ankle disorders: first metatarsal–phalangeal joint (MTPJ) extension [8, 11, 12], navicular drop (ND) test [10, 13] and forefoot varus angle (FVA) [9, 14]. Similarly, the Foot Health Status Questionnaire (FHSQ), Foot Function Index (FFI) and the American Academy of Orthopaedic Surgeons' Foot and Ankle Module (AAOS-FAM) are widely used in PROM in the assessment of a patient’s perception of foot–ankle disorders [7]. MoFAA and PROMs mentioned above have been used as outcome variable in clinical trials, demonstrating that these measurements are clinically relevant [15–21].

Previous studies have shown the usefulness of relating PROM and OCOM variables to different body regions, such as the back [3, 22] or knee [23], and to different population groups, such as subjects with intellectual disabilities [24, 25] or low back pain [3, 22].

To our knowledge, no study has been published that analyses and compares the relationship between the PROM and MoFAA (OCOM) and focuses on the assessment and monitoring of patients with metatarsal head pain. This study could be used in future to develop tools or protocols for assessment and monitoring patients with metatarsal head pain.

The present study had two objectives: first objective: to establish the relationship between foot–ankle PROM and MoFAA, which are commonly used in daily clinical practice for assessing and monitoring those patients with metatarsal head pain. Second objective of this study was to analyse how each of the three PROM selected are predicted by the three MoFAA selected. Our hypothesis was that the three questionnaires would correlate similarly with the three MoFAA during assessment.

## Methods

### Study design

The present cross-sectional study was developed in a clinical and human movement analysis laboratory. A combination of biomechanical and clinical data from questionnaires was collected between 1 February 2015 and 31 October 2015.

### Participants

**Fig. 1:**
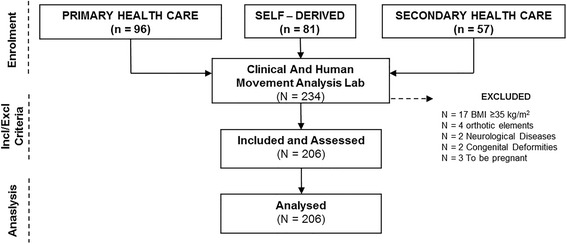
Flow diagram representing how many participants were recruited, included/excluded, and analysed

Two hundred and six patients participated in the present study. Inclusion criteria were: age between 18 and 65 years, metatarsal head pain. Exclusion criteria were: cognitive impairment, surgical intervention in lower limbs in the last year (because the normal biomechanics or position could be altered with a direct impact on the foot–ankle biomechanics/position [26–28]), congenital deformities and systemic or neuromuscular diseases that may affect lower limbs or foot posture, use of orthotic elements in lower extremities, body mass index (BMI) ≥35 kg/m^2^ or being pregnant. Figure 1 is a flow diagram from the recruitment to the analysis of the participants.

Informed consent was obtained from all individual participants included in the study. The study was conducted according to the Helsinki Declaration (Ethical Principles for Medical Research Involving Human Subjects) and was approved by the University of Malaga ethics committee.

### Measures of foot–ankle alignment (MoFAA)

Two blinded, independent reviewers with more than 15 years of experience in clinical podiatry, received a specific training to measure MoFAA included in the present study and performed each objective assessment. Each test was repeated three times for each investigator. Three MoFAA were recorded in this study. Firstly, the first MTPJ extension was assessed non–weight-bearing [8]. A goniometer was used and the medial midline of the hallux proximal phalanx and the medial midline of the first metatarsal were taken as reference and, as the apex of the angle, the first MTPJ. Secondly, the FVA was measured non–weight-bearing; the participant was placed in a prone position, and the subtalar joint was kept in its neutral position holding the hindfoot with one hand (through palpation of the talus head in the medial and lateral foot sides). The fixed arm of the goniometer was aligned parallel to the metatarsal heads (plantar aspect) (lateral to the 5° metatarsal head). The mobile arm of the goniometer was aligned perpendicular to the calcaneus’ bisectrix. The value of the FVA was the difference between 1st and 5th metatarsals position [29]. Finally, the ND test was performed, calculating the difference between the navicular height when the subject was sitting in a neutral-foot position and when the subject was standing in a relaxed-foot position [30]. In both, sitting and standing position, equal the distribution of the weight was equal on both legs.

The reliability of the three MoFAA have been established in previous studies, with values from 0.80–0.87 for the first MTPJ extension [8, 12], 0.98 for the FVA [9] and 0.91-0.94 for the ND test [31].

### Patient reported outcomes (PROM)

There were three questionnaires completed. The FHSQ was designed to measure the quality of life related to foot health [14]. This questionnaire uses four subscales (foot pain, foot function, footwear and general foot health) [32]. FHSQ scores range from 0 to 100, indicating the worst and best foot health status, respectively [32]. The Spanish version presented a reliability score of 0.93 [4] The FFI is a widely used tool for the assessment and management of patients with different origins of foot diseases, such as chronic problems, trauma and congenital or surgical correction [33]. It comprises a general index and three subscales that assess specific aspects of the foot: pain, disability and activity limitation [33]. The FFI score range between 0 (best foot function) to 100 (worst foot function) [33]. The Spanish version presents a reliability between 0.69 and 0.96 [33]. Finally, the AAOS-FAM is an instrument designed to evaluate patients’ perception of their foot health, and measure surgical outcomes [7]. It presents an overall value and two subscales: global foot and ankle scale and shoe comfort scale [7]. AAOS-FAM has a score range between 0 and 100 where 0 is the worst and 100 the best foot health [34] The Spanish version of the AAOS-FAM presented a reliability score between 0.79 and 0.99 [7].

### Procedure

Before starting the protocol, all participants’ anthropometric data were recorded (weight, height, BMI, age, gender and laterality). Then, participants completed the three PROM questionnaires in a randomized order. Once finished, each of the two blinded researchers (each of whom had more than 15 years of experience in clinical podiatry) took measures of the three MoFAA. Both researchers proceeded in the same way: They took measures on the participant’s right foot. Once all three measurements were done on the right foot, they removed all reference marks and started the measurements on the left foot. The same protocol, where both feet were alternate during measurement, was repeated three times. Similar than PROMs, all three MoFAA were measured in a randomized order to reduce bias from questionnaire fatigue.

### Statistical analysis

The data analysis was performed by an independent and blinded researcher. A descriptive analysis of the sample was performed. The Kolmogorov–Smirnov test was used to test for the normality of measurements. In addition, as a measure of control, MoFAA reliability (intra and inter-observer) was calculated through intraclass correlation coefficients (ICCs - CI 95%). The reliability was considered the consistency of MoFAA measures. Three measures were recorded for each MoFAA variable. Measures of reliability were stratified as follows: ICC ≤ 0.40 (poor), 0.60 > ICC > 0.40 (moderate), 0.80 > ICC ≥ 0.60 (good), ICC ≥ 0.80 (excellent) [35]. In addition, a correlation analysis between MoFAA and PROM was performed using Pearson’s coefficient, which was stratified into different levels: r ≤ 0.49 (poor), 0.50 ≤ r ≤ 0.74 (moderate), and r ≥ 0.75 (strong) [36]. In addition, linear regressions (where the dependent variable was each PROM index (or sub-scale) and independent variables [MoFAAs]) were calculated (including r^2^ and corrected r^2^). To develop the linear regressions, all MoFAAs (left and right side) were included as predictors in all regressions calculated (six variables in total for each model). In addition, standardised beta coefficients for each of the models were calculated. Normalised navicular drop test measures were used to calculate the relationship between MoFAA and PROMs variables (correlation analysis and linear regression analysis).

The Statistical Package for the Social Sciences (SPSS) (19.0 for Windows, Illinois, USA) was used to perform the statistical analysis.

## Results

Figure 1 represents how participants were recruited, and analysed. Descriptive analysis results of the sample of 206 participants (109 women and 97 men) can be observed (Table 1). All measurements were normally distributed. The average age of participants was 44.49 (±12.10) years. All participants were asked how many hours they spent standing per week (counting working time and leisure); the average was 45.76 (±6.91) hours. The mean values of other anthropometric variables, as well as MoFAA and PROM variables, can be observed in Table 1. The MoFAAs intra-observer reliability value (ICC–CI 95%) was of 0.942 (ND test), 0.957 (first MTPJ extension) and 0.919 (FVA). Inter-observer reliability (ICC–CI 95%) was 0.877, 0.891 and 0.861 for the ND test, first MTPJ extension and FVA, respectively.

**Table 1:** Sample descriptive of anthropometric, PROM and MoFAA data

			Mean	SD	Minimum	Maximum
Descriptive Variables	Age (years)		44.49	12.10	21	65
	Height (cm)		168	9.37	147	192
	Weight (kg)		76.7	6.24	42.40	103.30
	BMI (kg/m^2^)		27.13	2.11	19.57	33.54
	Standing time in a week (Hours)		45.76	6.91	20	65
MoFAA	Navicular Drop Test (mm)	Left	5.24	2.18	2.50	8.70
		Normalized (%)	11.04	2.55	4.30	14.15
		Right	5.31	2.23	2.30	8.60
		Normalized (%)	10.83	2.61	4.41	14.23
	1st MTPJ Extension (°)	Left	57.41	9.93	35	65
		Right	56.91	10.38	37	70
	Forefoot varus angle (°)	Left	5.57	6.51	-14	13
		Right	5.64	6.64	-16	13
PROM	AAOS FAM	AAOS FAM (0–100)	75.66	7.38	40	95
		Global Foot and Ankle Scale (0-100)	88.54	10.87	39	100
		Shoe Comfort Scale (0-100)	86.04	11.44	40	100
	FHSQsp	SHOE (0-100)	86.93	11.06	41.33	100
		FOOT FUNCTION (0-100)	79.82	14.79	31.25	100
		FOOT PAIN (0-100)	80.31	12.90	29.38	100
		GFH (0-100)	69.18	11.72	30.00	90
	FFI	FFI - Index 100-0)	88.48	11.41	37	100
		Pain (81-0)	70.12	8.24	38	81
		Disability (81-0)	68.83	6.05	32	81
		Activity limitations (35-0)	27.34	4.77	9	35
Laterality (left / right)			38 / 155			
N (women / men)			206 (109 / 97)			

*SD* Standard deviation, *BMI* Body mass index, *1st MTPJE* first metatarsalphalangeal joint, *AAOS FAM* AAOS foot and ankle module, *FHSQsp* Foot health status questionaire spanish version, *GFH* General foot health, *FFI* Foot functional index, *MoFAA* Measures of foot / ankle alignment, *PRO* Patients report outcome

Table 2 shows the correlation results for each PROM variable (scales and subscales) with respect to each MoFAA variable. Mostly correlations were significant, although the index value changed in each PROM used. Thus, in the AAOS-FAM questionnaire, correlation indices ranged from 0.243 (Shoe Comfort Scale–FVA right) and 0.686 (AAOS-FAM Index–first MTPJ extension right). In the FHSQ, correlation indices ranged from 0.282 (Shoe Comfort Scale–FVA right) to 0.643 (Foot Function–first MTPJ extension right). Finally, FFI correlation values ranged from 0.527 (Activity Limitations–FVA left) to 0.807 (FFI Index–first MTPJ extension right). The other correlation indices between PROM and MoFAA can be found in Table 2.

**Table 2:** Correlation levels of each MoFAA variable with each PROM (index and sub-escales)

			MoFAA						PROM										
			Navicular Drop Test		1st MTPJ Extension		Forefoot varus angle		Functional Foot Index				AAOS - FAM				FHSQsp		
			Right	Left	Right	Left	Right	Left	FFI Index	Pain	Disability	Activity limitations	AAOS-FAM Index	GFAS	SCS	Shoe	Foot Function	Foot Pain	GFH
MoFAA	Navicular Drop Test	Right	1																
		Left	0.961***	1															
	1st MTPJ Extension	Right	0.770***	0.740***	1														
		Left	0.719***	0.754***	0.912***	1													
	Forefoot varus angle	Right	0.728***	0.753***	0.667**	0.633**	1												
		Left	0.719**	0.755***	0.675**	0.636***	0.969***	1											
PROM	FFI	FFI Index	0.738***	0.731***	0.807***	0.793**	0.621***	0.629***	1										
		Pain	0.719***	0.704***	0.784**	0.748**	0.597**	0.603**	0.854***	1									
		Disability	0.714***	0.694**	0.791***	0.760**	0.608***	0.611***	0.827***	0.904***	1								
		Activity limitations	0.656**	0.661***	0.673**	0.690**	0.533**	0.527**	0.801***	0.842***	0.912***	1							
	AAOS FAM	AAOS FAM Index	0.662***	0.670**	0.686**	0.681***	0.527**	0.521**	0.837***	0.807**	0.826***	0.758***	1						
		GFAS	0.651**	0.657*	0.686**	0.666***	0.517*	0.508**	0.799***	0.719***	0.804***	0.721**	0.977***	1					
		SCS	0.299	0.309	0.345	0.320	0.243	0.251	0.474*	0.429*	0.500**	0.399	0.477**	0.277	1				
	FHSQsp	Shoe	0.367	0.380	0.451**	0.439*	0.282	0.303	0.610***	0.540**	0.579**	0.510*	0.545**	0.428*	0.690**	1			
		Foot Function	0.579**	0.569***	0.606*	0.609**	0.454**	0.479**	0.700***	0.633**	0.619**	0.543***	0.622***	0.605**	0.310	0.413*	1		
		Foot Pain	0.592**	0.579**	0.643**	0.640**	0.484**	0.497**	0.745***	0.687***	0.673***	0.631**	0.625***	0.653**	0.304	0.435**	0.906***	1	
		GFH	0.502*	0.491*	0.540**	0.534**	0.374	0.395*	0.653***	0.607**	0.611***	0.528***	0.596**	0.598***	0.219	0.384	0.780***	0.847***	1

*1st MTPJE* first metatarsalphalangeal joint, *AAOS FAM* AAOS foot and ankle module, *GFAS* Global Foot and Ankle Scale, *SCS* Shoe comfort scale, *FHSQsp* Foot health status questionaire spanish version, *GFH* General foot health, *FFI* Foot functional index, *MoFAA* Measures of foot / ankle alignment, *PRO* Patients report outcome

Significance * ≤ 0.05, ** ≤ 0.005, *** ≤ 0.001

Table 3 shows the regression values, where dependent variables were the PROM (index and subscales); independent variables were the MoFAA. Thus, regression indices (R^2^ corrected) ranged between 0.117 (AAOS-FAM–Shoe Comfort Scale) and 0.701 (FFI Index). In Table 4, the standardized beta coefficients of each independent variable in the regression of dependent variables are presented. The independent variable that contributed most to the values of regression is the first MTPJ extension, followed by the ND test and the FVA. In addition, the dependent variables had lower regression index values than those that were related to footwear, which made the greatest contribution to FVA.

**Table 3:** Results of multiple regressions analysis, PROM were the dependent variables and MoFAA were the independent variables

	Dependent variable (Model)	R	R^2^	R^2^ corrected	Sig.
AAOS-FAM	AAOS-FAM Index	0.732	0.536	0.521	0.000
	Global Foot and Ankle Scale	0.694	0.481	0.465	0.000
	Shoe Comfort Scale	0.380	0.144	0.117	0.000
FHSQsp	Shoe	0.466	0.217	0.192	0.000
	Foot function	0.657	0.542	0.499	0.000
	Foot pain	0.680	0.563	0.502	0.000
	GFH	0.577	0.333	0.311	0.000
FFI	FFI Index	0.843	0.710	0.701	0.000
	Pain	0.724	0.637	0.619	0.000
	Disability	0.709	0.629	0.606	0.000
	Activity limitations	0.638	0.587	0.553	0.000

*AAOS FAM* AAOS foot and ankle module, *FHSQsp* foot health status questionaire spanish version, *GFH* General foot health, FFI Foot functional index

**Table 4:** Standardized Beta coefficients of each independent variable (MoFAA) for each dependent variable (PROM index and sub-scales)

	Dependent variable (Model)	Navicular Drop Test		1st MTPJ Extension		Forefoot varus angle		R^2^ corrected
		Right	Left	Right	Left	Right	Left	
AAOS-FAM	AAOS-FAM Index	0.205	0.153	0.449	0.422	0.096	0.145	0.521
	Global Foot and Ankle Scale	0.223	0.123	0.179	0.280	0.123	0.195	0.465
	Shoe Comfort Scale	0.135	0.039	0.270	0.066	0.206	0.330	0.117
FHSQsp	Shoe	0.042	0.071	0.277	0.148	0.322	0.249	0.192
	Foot function	0.139	0.270	0.505	0.414	0.446	0.459	0.499
	Foot pain	0.209	0.322	0.501	0.409	0.277	0.262	0.502
	GFH	0.202	0.222	0.435	0.233	0.400	0.353	0.311
FFI	FFI Index	0.327	0.232	0.463	0.451	0.112	0.168	0.701
	Pain	0.307	0.218	0.432	0.493	0.103	0.155	0.619
	Disability	0.299	0.211	0.439	0.428	0.097	0.151	0.606
	Activity limitations	0.246	0.186	0.410	0.403	0.114	0.106	0.553

*1st MTPJE* first metatarsalphalangeal joint, *AAOS FAM* AAOS foot and ankle module, *FHSQso* Foot health status questionaire spanish version, *GFH* General foot health, *FFI* Foot functional index

## Discussion

Based on the results, a relevant finding of the present study is that the MoFAA variables are most highly correlated with the FFI Index (*r* = 0.621-0.807). In addition, FFI index has the highest corrected R^2^ (0.701) in multivariable regression out of all the PROMs tested.

### MoFAA –PROM relationship

Among the three questionnaires used in this study, the MoFAA had the greatest influence on the FFI (R^2^ corrected 0.701), followed by the AAOS-FAM (R^2^ corrected 0.521) (Table 3), while the FHSQ did not show a specific, general index that included all subscales. This greater influence of MoFAA variables on the PROMs could mean that, in the FFI, the questions (wondering and wondering how) included patients’ self-reported results of the objective assessment. Analysing the results of the linear regression analysis MoFAAs did not determine equally the subscales of each questionnaire (Table 4). In addition, regarding the way in which each MoFAA variable correlated with PROM variables, the subscales related to pain and function showed the highest correlation value (FFI_Pain_: 0.619, FFI_Disability_: 0.606, FFI_Activity_Limitation_: 0.553, FHSQ_Foot_Pain_: 0.502, FHSQ_Foot_Function_: 0.499) (Table 4). On the other hand, subscales that included specific questions about shoes had the lowest correlation with MoFAA; none of the questionnaires that included a shoe subscale reached standardised beta coefficient values higher than 0.200 (AAOS-FAM_Shoe_Comfort_Scale_: 0.117, FHSQ_Shoe_: 0.192) (Table 3).

### MoFAA: individual relationship analysis

Regarding the degree of influence that each of the MoFAA variables had when correlating with PROM and subscales, the first MTPJ extension was the most determining MoFAA variable in the PROM (FFI and AAOS- FAM) and in the subscales that assessed pain and function; standardized beta coefficient values ranged from 0.403 (FFI_Activity_Limitation_) to 0.505 (FHSQ_Foot_Funtion_). The significance of the first MTPJ mobility for activities of development, such as walking or running, was shown in previous studies [8, 11, 13, 37]. However, this was the first study that related the mobility of the first MTPJ to the patient's subjective perception of function and pain, showing, once again, that the first MTPJ played an essential role in health status, function and foot pain perceived by the patient.

Although both the ND test and the FVA were significant with the patient in a static position [38, 39], in ambulation [38] and even at the onset of musculoskeletal disorders [38, 39], the influence of these two MoFAA variables was perceived to have less relevance by patients.

### Applicability of the relationship between MoFAA and PROM foot–ankle

To our knowledge, this is the first study that analyses the relationship between MoFAA and PROM used for patients’ assessment and management of metatarsal head pain; thus, it was not possible to compare the results of similar studies.

However, recent studies conducted on other body regions showed high applicability in finding a relationship between MoFAA and PROM variables, such as to create a discriminatory rate in people with chronic, non-specific low back pain [3], to be used as a strength predictor index in the rehabilitation processes in the ACL [24], to find an association between disability and mobility in patients with lumbar symptomatic spondylolisthesis [23] or to associate, in subjects with intellectual disabilities, physical tests with dependency levels [25, 40]. In the same line, the results obtained in this study, in which a moderate to strong relationship between MoFAAs and PROMs was observed, could be used in future studies to develop tools for assessment and monitoring patients with metatarsal head pain.

FVA measurements were taken considering the alignment of the five metatarsals with respect to the rearfoot [29]. This methodology differs with respect to the classic Rootian methodology, which focuses on examination of the alignment of metatarsals 2–4 in relation to the rearfoot. It is important to consider when interpreting the results that the levels of correlation of this variable (FVA) with respect to other variables considered in this study could be subject to change if it has been opted to use the classic Rootian methodology.

### Reliability

As a control strategy for the MoFAA, the stability of the measurements was calculated through ICC (CI 95%). The results showed excellent reliability in the three MoFAA selected in the present study. The stability of intra-observer measures was 0.942 (ND test), 0.957 (first MTPJ extension) and 0.919 (FVA), which was consistent with previous studies that reported values of 0.91–0.97 (ND test) [10, 13, 41], 0.65–0.975 (first MTPJ extension) [23, 37] and 0.98–0.99 (FVA) [9, 14]. In the same way, the stability of inter-observer measures was 0.877, 0.891 and 0.861 for the ND test, the first MTPJ extension and the FVA, respectively. These results were consistent or slightly higher than previous studies, which showed values of 0.43–0.97 (ND test) [10, 13, 31], 0.693–0.953 (first MTPJ extension) [23. 28] and 0.92 (FVA) [9]. All MoFAA variables reached a value of stability measure greater than 0.9 (intra-observer) and 0.8 (inter-observer), which can be considered valid for measurements performed with a goniometer [42]. Both researchers were instructed in the same way to assess each MoFAA. In addition, they were practicing together and they used only one decimal place for measurement. All these aspects could influence positively to the observed results.

### Strengths and weaknesses

This study analysed the cross-sectional relationship between MoFAA and PROM, while observing the influence of MoFAA on PROM correlation using a cross-sectional study design. However, future longitudinal studies could analyse the variation of the variables over time, as well as their relationships, the inclusion of subjects between 18-65 represents a heterogeneous group and represents a potential source of bias that should be considered, for this reason, future studies should increase the sample and divided it in different age-group (young adults, middle-age adults and older adults (for example) to considered eventual differences between participants due their age. The participant sample (*n* = 206) was insufficient to analyse this patient profile by dividing the sample in subgroups based on particular characteristics of the sample, such as body mass index, pregnancy, use of orthotic elements, etc. For this reason, these results may not be generalizable to patients with a specific characteristic (obesity, to be pregnant, etc.) subjects with foot pain Future study should be design to analyse the relationship between MoFAA and PROMs considering specific characteristics of the sample. The absence of biological plausibility and with standard information about measures scores (mean and SD), there was a risk of false positives. To minimize this potential risk, the authors have increased the sample until the final 206 participants and have used a confidence interval of 95% for correlation analysis. Finally, the use of any medication could affect specific pain questions of the PROMs, and it could be important to interpret the results of the specific pain question.

## Conclusion

The MoFAA correlated between moderately to strongly with the foot–ankle PROM selected. MoFAA demonstrated the highest influence on FFI index, which could mean that FFI index more effectively represents the relationship between objective physical state of the patient’s feet and the patient’s perception about the health status of his/her feet. The influence level was higher when patients with metatarsal head pain were asked about foot health status, pain and function; however, the influence level was poor when the patient was asked about shoe aspects. Finally, the MoFAA variable achieved the highest correlation value: first, the MTPJ extension, followed by the ND test and the FVA. The results obtained in this study could be used in future studies to develop tools for assessment and monitor patients with metatarsal head pain.

## Abbreviations

AAOS-FAM: American academy of orthopaedic surgeons-foot and ankle module
BMI: Body mass index
FFI: Foot function index
FHSQ: Foot health status questionnaire
FVA: Forefoot varus angle
MoFAA: Measures of foot–ankle alignment
MTPJ: First metatarsal–phalangeal joint
ND: Navicular Drop
OCOM: Objective clinical outcome measures
PROM: Patient-reported outcome measures
